# From Gym to ICU: A Case of Exertional Rhabdomyolysis‐Induced Acute Kidney Injury in a Healthy Young Athlete

**DOI:** 10.1002/ccr3.71130

**Published:** 2025-10-08

**Authors:** Musawer Khan, Asif Ullah Khan, Ayesha Ahmad, Areeba Ali Khan, Muhammad Naseer, Ghazal Ishaque, Ahmad Aneeq, Alamzaib Khan, Kamil Ahmad Kamil

**Affiliations:** ^1^ Internal Medicine Department Combined Military Hospital Quetta Pakistan; ^2^ Khyber Medical University Peshawar Pakistan; ^3^ Services Institute of Medical Sciences Lahore Pakistan; ^4^ Shaeed Mohtarma Benazir Bhutto Medical College Karachi Pakistan; ^5^ Quaid e Azam Medical College Bahawalpur Pakistan; ^6^ Internal Medicine Department Mirwais Regional Hospital Kandahar Afghanistan

**Keywords:** acute kidney injury, CPK, exercise‐induced rhabdomyolysis, hemodialysis, young athlete

## Abstract

Exertional rhabdomyolysis can rapidly progress to acute kidney injury requiring dialysis, even in young healthy athletes. Delayed presentation and inadequate hydration increase risk. Clinicians should suspect rhabdomyolysis in patients with myalgia, dark urine, or oliguria after strenuous exercise, and initiate early hydration and electrolyte monitoring to prevent severe complications.

## 1 Introduction

Rhabdomyolysis is a clinical syndrome characterized by skeletal muscle breakdown and release of intracellular contents such as myoglobin and creatine phosphokinase (CPK) into the circulation [1]. It can result from trauma, ischemia, infections, toxins, medications, metabolic or endocrine disorders, and strenuous exercise [2].

Exertional rhabdomyolysis is increasingly recognized in athletes, military recruits, and recreational gym users, typically presenting with muscle pain, fatigue, and dark urine. While most cases are mild, 10%–30% may develop acute kidney injury (AKI), which significantly increases morbidity and mortality [2].

This case is presented to highlight the risk of delayed presentation leading to dialysis‐requiring AKI in a previously healthy young athlete, and to underscore the importance of early recognition, hydration, and electrolyte monitoring in preventing severe complications.

## 2 Case Presentation

A 22‐year‐old previously healthy male with a history of regular intense gym workouts presented to the emergency department with severe bilateral flank pain and persistent vomiting for the last 10 days, along with reduced urine output (oliguria) and dark‐colored urine. He also reported intermittent fever for 2 weeks. There was no history of trauma, substance use, or comorbid illness.

On arrival, the patient appeared dehydrated, acutely ill, but fully conscious and oriented (GCS 15/15). Vital signs were stable (BP 120/90 mmHg, pulse 74 bpm, SpO_2_ 96% on room air). Cardiovascular and respiratory examinations were unremarkable. Abdominal examination revealed a soft, non‐tender abdomen. A blanching rash was noted over the trunk.

**FIGURE 1:**
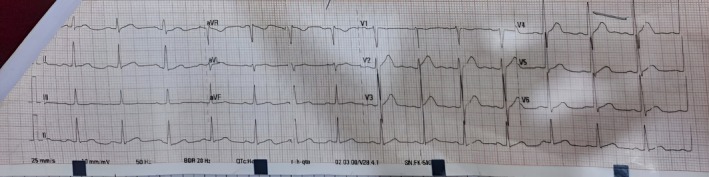
12‐lead ECG on admission showing sinus rhythm with T wave abnormalities and QT interval prolongation, suggestive of evolving electrolyte disturbance in the context of rhabdomyolysis.

Initial laboratory investigations revealed markedly elevated creatine phosphokinase (CPK 80,064 U/L), severe renal impairment (serum creatinine 1828 μmol/L, urea 70.4 mmol/L, BUN 88 mg/dL), and hyponatremia (Na 121 mmol/L). He also had metabolic acidosis (pH 7.24, HCO_3_^−^ 12 mmol/L) and hypocalcemia (Ca 1.9 mmol/L). An ECG at presentation showed sinus rhythm with diffuse T wave abnormalities and a prolonged QT interval rather than true “peaked” T waves (Figure 1). While serum potassium was normal (4.8 mmol/L), these repolarization changes raised concern for evolving electrolyte imbalance in the setting of rhabdomyolysis and prompted intensive cardiac monitoring. Table 1 summarizes admission labs.

**TABLE 1:** Laboratory investigation on the day of admission.

Labs variable	Result reading on admission day	Reference range
Hemoglobin, g/dL	13.8	13.0–17.0 g/dL
White cell count, 10^9^/L	8.5	4.0–11.0 × 10^9^/L
Platelets count, 10^9^/L	284	150–400 × 10^9^/L
Creatinine, μmol/L	1828	71–115 μmol/L
Creatinine phosphokinase (CPK), U/L	80,064	M: 46–171, F: 34–145
Blood urea nitrogen (BUN), mg/dL	88	06–20
Glycosylated hemoglobin	5.2%	6.7%
Urea, mmol/L	70.4	2.1–7.1
Sodium (Na), mmol/L	121	136–149
Potassium (K), mmol/L	4.8	3.5–5.0
CRP‐C reactive protein	02	< 6 mg/L
Serum alkaline phosphatase	83	40–305 IU/L
Calcium (adults), mmol/L	1.9	2.1–2.65 mmol/L
Phosphorus (adults), mmol/L	0.83	0.81–1.62 mmol/L
Albumin, g/dL	36	35–50 g/L
Serum ALT	113	0–42 U/L
Serum bilirubin	05	0‐17 μmol/L
Prothrombin time	16/14	14/14
PTTK	38/32	32/32
INR	1.20	1
D‐dimer	> 200 < 400	< 200 ng/mL
Thyroid function test	Normal	Normal
pH	7.24	7.35–7.45
PCO2, mmHg	21	35–45 mmHg
PO2, mmHg	116	80–110 mmHg
HCO3, mmol/L	12	22–28 mmol/L
SAT, %	97%	95%–100%
Serum LDH	479	125–220 U/L
Antibody to hepatitis C virus	Negative	Negative
Hepatitis B surface antigen	Negative	Negative
RA factor	Negative	Negative
Anti‐dengue virus antibodies	Negative	Negative
Malarial parasite	Negative	Negative
p‐ANCA	Negative	Negative
c‐ANCA	Negative	Negative
RF	Negative	Negative

The patient was started on intravenous 0.9% normal saline at 250 mL/h with sodium bicarbonate infusion to maintain urine alkalinization. Serum sodium was monitored every 6 h, showing gradual correction (trend added in Table 2). Despite aggressive hydration (≈5 L within 24 h), urine output did not improve, and he remained oliguric. Given worsening azotemia, metabolic acidosis, and uremic symptoms, the patient was shifted to the Intensive Care Unit for closer monitoring and renal replacement therapy.

**TABLE 2:** Comparison of laboratory testing results obtained on admission day, during treatment, on the day of discharge, and at 2 weeks follow‐up.

Variables	Day 1 (hospital admission)	Day 2	Day 3	Day 4	Day 5	Day 6	Day 10 hospital discharge	Follow‐up after 2 weeks of discharge
Creatinine, μmol/L	1828	1886	955	267	333	263	83	91
Urea, μmol/L	70.4	89	40	17	19	15	6.2	5.5
Blood urea nitrogen (BUN), mg/dL	88	91	55	23	21	17	18	11
Creatinine phosphokinase (CPK), U/L	80,064	81,059	39,652	1700	210	126	140	134
Lactate dehydrogenase (LDH), adult, U/L	479	490	369	263	229	117	140	128
Serum sodium (Na, mmol/L)	121	126	134	137	138	140	140	142

**FIGURE 2:**
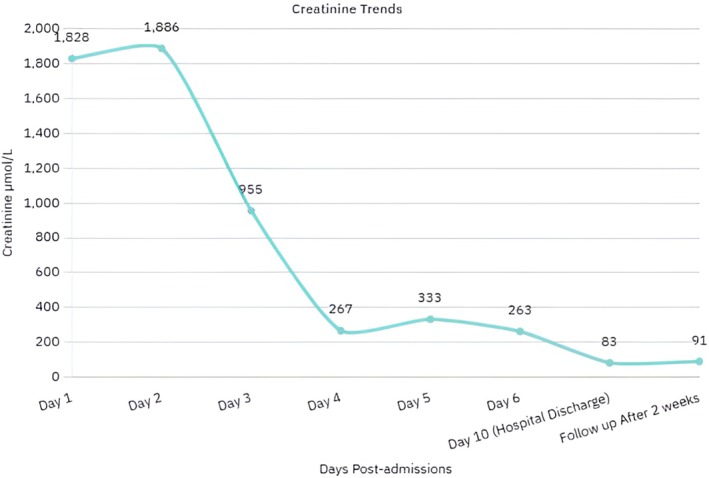
Trend of serum creatinine levels during the first 2 weeks post‐admission. A peak was observed on day 2, followed by a progressive decline and normalization by the end of the second week, indicating early renal recovery.

**FIGURE 3:**
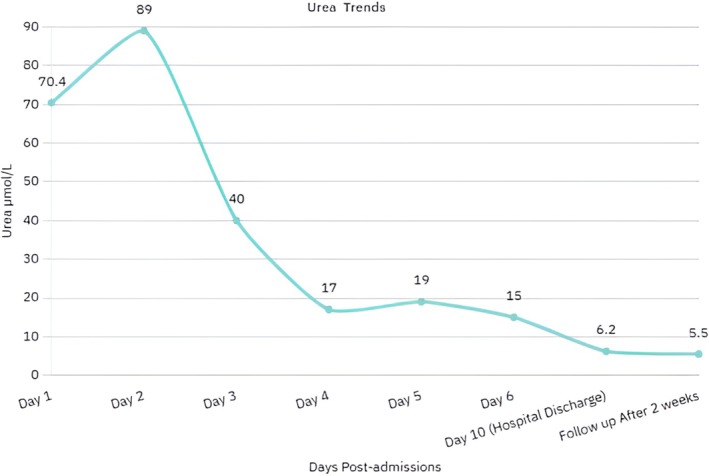
Serum urea levels peaked on day 2 and normalized by day 14, indicating early renal recovery.

Hemodialysis was initiated on day 2 via a right femoral central venous catheter inserted under ultrasound guidance using the Seldinger technique. Dialysis was performed for 2 h initially without ultrafiltration. Over the next 3 sessions, there was progressive clinical improvement with declining creatinine and urea levels (Figures 2 and 3, Table 2).

Additional investigations (thyroid function, viral hepatitis serology, p‐ANCA/c‐ANCA, rheumatoid factor, malaria, and dengue serology) were performed to exclude autoimmune, infectious, and metabolic causes of rhabdomyolysis; all were negative.

### 2.1 Follow Up

- First month: After discharge, the patient showed remarkable renal recovery, with serum creatinine normalizing to the range of 90–110 μmol/L by the fourth week, by which time evidence of rhabdomyolysis resolution was seen in the form of a substantial reduction in creatine phosphokinase (CPK) levels from more than 80,000 U/L at admission to baseline levels of less than 200 U/L. There was a biochemical improvement but the patient experienced chronic fatigue throughout this phase.
- 3rd month: By the 3rd month due to supervised physiotherapy, patient reported substantial improvement in fatigue and marked restoration of muscle strength. Laboratory tests confirmed stable renal function (creatinine ~100 μmol/L, eGFR > 60 mL/min/1.73 m^2^) and no signs of proteinuria or electrolyte imbalances. There were no signs of rhabdomyolysis recurrence.
- 6th month: The patient remained asymptomatic and no residual kidney dysfunction was noted. His CPK and LDL levels remained within normal limits. A final renal ultrasound showed no abnormalities hence ruling out kidney damage. The patient was instructed to have a balanced exercise program and stay well hydrated to promote continued recovery and prevent recurrence.

## 3 Discussion

Exertional rhabdomyolysis has become increasingly recognized among athletes, military recruits, and recreational gym members, with incidence rising in otherwise healthy individuals [3, 4]. Our case demonstrates how delayed recognition of exertional rhabdomyolysis can progress to dialysis‐requiring acute kidney injury (AKI), despite the patient being young, previously fit, and without comorbidities.

The patient presented late, with more than 10 days of persistent vomiting and flank pain, which likely contributed to dehydration and worsened renal injury. This highlights the critical role of early hydration and timely clinical suspicion. Although his creatine phosphokinase (CPK) levels were markedly elevated (80,064 U/L), the clinical presentation of rhabdomyolysis can vary from asymptomatic increases in CK to potentially life‐threatening complications; acute kidney injury (AKI) is still one of its most dreaded complications [5]. The mortality risk is greatly enhanced when AKI occurs, with mortality rates ranging from 10% to as much as 50% in severe cases necessitating renal replacement therapy [6]. Apart from renal impairment, rhabdomyolysis may result in life‐threatening systemic complications, such as electrolyte imbalance in the form of hyperkalemia, which can induce lethal cardiac arrhythmias. Sudden cardiac death, although uncommon, has been described especially when hyperkalemia or hypocalcemia is not considered or not treated [7, 8]. This supports evidence demonstrating that the severity of renal injury does not always correlate with the degree of CK elevation, but the duration of muscle breakdown, dehydration, and delayed treatment determine renal damage [9]. Rather, the duration of muscle breakdown, delayed fluid resuscitation, and electrolyte imbalances determine outcomes.

Electrolyte disturbances are common in rhabdomyolysis. Our patient developed severe hyponatremia (Na 121 mmol/L) and hypocalcemia. These abnormalities can contribute to fatigue, nausea, and altered sensorium, but were corrected gradually with fluid therapy. Importantly, rhabdomyolysis in this case was not precipitated by hyponatremia, but rather by intense exercise and inadequate hydration.

Electrocardiographic changes are another important clinical clue. Although the patient's serum potassium was normal, his ECG showed QT prolongation and nonspecific T wave abnormalities. This finding underscores that electrical alterations may precede measurable electrolyte derangements, warranting early monitoring and intervention in suspected rhabdomyolysis.

### 3.1 Prevention and Awareness

This case emphasizes the need for preventive measures, particularly in high‐intensity athletes. Adequate hydration, progressive training protocols, supervision during exercise, and mandatory rest periods are essential strategies to reduce the risk of exertional rhabdomyolysis. Clinicians should maintain a high index of suspicion in patients presenting with nonspecific symptoms (vomiting, myalgia, oliguria) after strenuous exercise.

### 3.2 Limitations

This report describes a single case, and we cannot rule out contributing factors such as undocumented dehydration before admission. Nonetheless, the clinical course provides a strong teaching point for both clinicians and athletes.

## 4 Conclusion

This case is illustrative of the potentially devastating outcomes of exertional rhabdomyolysis, even in healthy, young patients with no comorbidities. In spite of moderate elevation of CPK levels, our patient ended up having a life‐threatening acute kidney injury requiring hemodialysis, reaffirming the truth that delayed presentation and poor hydration can drastically complicate outcomes. Clinicians must have a high level of suspicion for rhabdomyolysis in patients who have nonspecific symptoms like vomiting, myalgia, or decreased urine output, particularly in the setting of recent vigorous exercise.

## Author Contributions

**Musawer Khan:** conceptualization, data curation, investigation, supervision, writing – original draft. **Asif Ullah Khan:** resources, validation, writing – review and editing. **Ayesha Ahmad:** investigation, resources, supervision, validation, writing – review and editing. **Areeba Ali Khan:** investigation, resources, supervision, writing – review and editing. **Muhammad Naseer:** validation, writing – review and editing. **Ghazal Ishaque:** writing – review and editing. **Ahmad Aneeq:** methodology, validation, writing – review and editing. **Alamzaib Khan:** methodology, visualization, writing – review and editing. **Kamil Ahmad Kamil:** investigation, methodology, visualization, writing – original draft, writing – review and editing.

## Ethics Statement

All procedures performed during the study (case report) were in accordance with ethical standards of the Faculty of Medicine, Institutional Ethical Review Board (IERB) CMH Quetta.

## Consent

Written informed consent was obtained from the patient for publication of this case report.

## Conflicts of Interest

The authors declare no conflicts of interest.

## Data Availability

The authors have nothing to report.
